# Multi-Omics Reveals the Effects of Processing on Chemical Composition of Different Vegetable Oils

**DOI:** 10.3390/foods15152738

**Published:** 2026-08-04

**Authors:** Xuheng Nie, Shidong Lv, Xin Wang, Xuefeng Chen, Yunman Wen, Shuiyan Yang, Chao Liu

**Affiliations:** 1 College of Biology and Food Engineering, Qujing Normal University, Qujing 655011, China; niekon2014@163.com; 2 Yunnan Provincial Academy of Food and Oil Sciences, Kunming 650033, China; ynwx@sina.com (X.W.); 13708710327@163.com (X.C.); wpt212@sina.com (Y.W.); 3 Kunming Grain Oil and Feed Products Quality Inspection Center, Kunming 650118, China; shidonglv@163.com

**Keywords:** soybean oil, rapeseed oil, multi-omics, chemometric analysis, processing

## Abstract

Soybean oil (SO) and rapeseed oil (RO) are important vegetable oils widely used in food and food preparation. However, the multi-omics profile changes in these edible oils remain unclear. This study investigated the effects of varieties and processing on oil compounds. In total, 1033 widely targeted metabolites, 349 lipid compounds, and 157 volatile organic metabolites were identified in the samples. Chemometric analysis revealed that the number of differential metabolites was 664, 857, 204, and 812 in SO_1stg_ vs. RO_1stg_, SO_3rdg_ vs. RO_3rdg_, SO_3rdg_ vs. SO_1stg_, and RO_3rdg_ vs. RO_1stg_, respectively. Notably, upregulated differential metabolites accounted for 92.73% of the total differential metabolites in the comparison of RO_3rdg_ vs. RO_1stg_, and 79.41% in the comparison of SO_3rdg_ vs. SO_1stg_. This study provides data support and a theoretical basis for constructing regulatory systems in the edible plant oil (EPO) and fat industry.

## 1. Introduction

Edible plant oil (EPO) is a vital source of dietary fat and fat-soluble nutrients essential for human health worldwide. It is derived from the seeds, pulps, fruits, and plumules of certain plants [1]. Rapeseed oil (RO) and soybean oil (SO) are the two most widely consumed EPOs globally due to their high production volumes and relatively balanced fatty acid compositions [2]. These oils are commonly used in food and food preparation and are primarily composed of triglycerides and typically remaining liquid at room temperature. According to the latest report from the United States Department of Agriculture, global SO production exceeded 60 million tons during the 2022/2023 crop year, accounting for nearly 30% of the total world EPO output [3]. In 2023, China’s edible oil consumption reached 40.96 million tons, with RO production at 5.27 million tons, SO production at 0.066 million tons, RO imports at 23.56 million tons, and SO imports at 0.4 million tons [4]. The nutritional quality, authenticity, and safety of these oils are directly linked to consumer rights and public health.

The refining process significantly impacts nutritional quality. To achieve clear color, neutral flavor, and stable oxidation properties in high-quality oil, refining EPO is essential to remove free fatty acids, phospholipids, pigments, odor-causing substances, and impurities from crude oil. Due to variations in processing, EPO is typically classified into first-grade, second-grade, and third-grade categories. However, during processing, bioactive compounds are lost, leading to a decline in nutritional quality. For example, the deodorization process substantially reduces phytosterol levels [5]. Total phenol content decreases by approximately 98% during the decolorization and deodorization of RO [5,6]. Roasting also affects the nutritional value and palatability of the resulting oil [7]. Overall, 10–36% total tocopherols, 6–52% of total sterols, and 93–98% of polyphenols are destroyed during refining [8]. Currently, few studies have examined the impact of processing on EPO quality from a multi-omics perspective.

RO is widely regarded as one of the healthiest EPO [9] and is preferred due to its favorable fatty acid composition [10,11]. It contains several essential unsaturated fatty acids, including oleic acid, linoleic acid, and linolenic acid, which contribute to its nutritional and health benefits [11,12]. Its well-balanced fatty acid profile can help alleviate chronic conditions such as cardiovascular and cerebrovascular disorders, cancer, and diabetes, primarily by regulating cholesterol and triglyceride levels and improving blood cell viscosity [13]. SO is the leading EPO in terms of both global production and consumption. It is characterized by high yield, low cost, and affordability. SO is rich in polyunsaturated fatty acids, comprising 55–60% of its content and linoleic acid as the predominant component, accounting for approximately 50–55% [14]. According to the latest edible oil market report (Issue 32, 2025), the price of first-grade SO (SO_1stg_) is approximately 8600 yuan per ton, while first-grade RO (RO_1stg_) is about 10,000 yuan per ton. One common form of adulteration involves mixing low-priced SO with high-priced RO [15]. Therefore, minimizing nutrient loss in SO and RO caused by excessive processing is crucial.

Cumulative results and empirical findings have been established to detect and quantify components of edible oils. The identification of compounds in EPO is shifting from targeted analysis to non-targeted comprehensive analysis. Current research primarily focuses on employing high-resolution, and high-throughput modern analytical techniques to obtain global chemical profiles of edible oils [16,17]. For example, gas-chromatography coupled with mass spectrometry (GC-MS) has been used to detect adulteration in camellia oil [16]. Other studies have utilized high-resolution mass spectrometry to investigate adulteration in SO and peanut oil, using isoflavones and resveratrols as indicators [18]. Relevant research has also differentiated and analyzed extra virgin olive oil by integrating three-dimensional fluorescence with two-dimensional near-infrared spectral data [19]. Multisource data fusion analysis is another effective method for food identification [20]. By integrating multiple data sources such as stable isotopes, elemental composition, and volatile compounds, the geographic origin of soybeans has been traced [21]. Metabolomics has been widely applied in tracing the origin of plant-based foods, identifying cultivated varieties, and monitoring processing effects [22]. Additionally, lipidomics and metabolomics techniques have been used to identify biomarkers influencing fat deposition in the breast muscles of broiler chickens at 42 and 180 days of age [23]. However, the application of multi-omics technologies for quality evaluation of edible oils remains limited. Therefore, we analyzed the metabolite profiles of RO_1stg_, the third-grade-RO (RO_3rdg_), SO_1stg_, and the third-grade-SO (SO_3rdg_) from the perspectives of metabolomics, lipidomics, and volatileomics. This approach will deepen our understanding of how processing affects the components of edible oils, and provide data and a theoretical basis for detecting adulteration and forgery in edible oils.

This study was primarily designed to address the following objectives: (1) the metabolic variations in RO_1stg_, RO_3rdg_, SO_1stg_, and SO_3rdg_; (2) the impact of processing on the components of RO and SO; and (3) potential biomarkers distinguishing RO from SO. We hypothesized that (1) RO and SO exhibit different metabolomics profiles, and (2) processing significantly affects the metabolites of both RO and SO. Our research provides a theoretical foundation for moderate processing, quality evaluation, and adulteration detection of edible oils. Therefore, this study holds considerable theoretical and practical significance.

## 2. Materials and Methods

### 2.1. Chemicals and Reagents

Hydrochloric acid was purchased from Xinyang Chemical Reagent (Xinyang, China). HPLC-grade methanol (MeOH), ethanol (EtOH), and acetonitrile (ACN) were purchased from Merck (Darmstadt, Germany). Butylated hydroxytoluene (BHT) was supplied by Aladdin (Shanghai, China). Acetone was supplied by Sinopharm (Shanghai, China). Methyl tert-butyl ether (MTBE) was supplied by CNW (Shanghai, China). Sodium chloride (NaCl) was supplied by Rhawn (Shanghai, China), and potassium hydroxide (KOH) was supplied by Hushi (Shanghai, China).

### 2.2. Plant Materials and Experimental Design

We purchase RO and SO from a local oil processing plant. These edible oils are produced by physically pressing rapeseed and soybeans, followed by moderate refining processes applied to three different batches in oil processing plant. The initially processed RO undergoes degumming, deacidification, decolorization, and deodorization treatments to produce RO_1stg_. Another batch of initially processed RO undergoes only degumming and deacidification to produce RO_3rdg_; the RO_3rdg_ does not undergo deep decolorization or deodorization. The initially processed SO undergoes degumming, deacidification, decolorization, deodorization, and winterization treatments to remove gums, free fatty acids, pigments, volatile odor substances and wax, resulting in the SO_1stg_. The winterization and dewaxing steps are unique to SO refining. Another batch of initially processed SO undergoes only degumming and deacidification to produce SO_3rdg_; the SO_3rdg_ does not undergo deep decolorization or deodorization. Samples of SO_1stg_, SO_3rdg_, RO_1stg_, and RO_3rdg_ were collected immediately after processing, with each sample approximately 400 mL in volume. The samples were promptly stored at −80 °C until analysis.

### 2.3. Sample Preparation and Extraction

#### 2.3.1. Widely Targeted Metabolomics Preparation and Extraction

Remove the samples from the −80 °C freezer and allow them to thaw. Vortex the samples for 30 s. Transfer 500 μL of each sample using a pipette and mix with 1000 μL of 70% methanol containing the internal standard. For samples smaller than 500 μL, adjust the volume to maintain a 1:2 dilution ratio accordingly. Vortex the mixture for 3 min to ensure thorough mixing, then incubate overnight at 4 °C. The following day, vortex the samples again for 3 min, then sonicate for 30 s to eliminate foam. Centrifuge the samples at 12,000 rpm and 4 °C for 3 min. Carefully remove 800 μL of the organic phase, filter it through a 0.22 μm microporous membrane, and transfer the filtrate into an injection vial for subsequent UPLC-MS/MS analysis. A mixture of SO and RO extracts was used as a quality control (QC) sample and analyzed in triplicate.

#### 2.3.2. Lipid Compounds Preparation and Extraction

A 10 mg sample was placed into a 2 mL centrifuge tube. One milliliter of lipid complex solution (acetonitrile: isopropanol, 1: 1) was added and mixed thoroughly by vortexing for 1 min. Then, transfer 10 μL of the resulting diluent into a new tube, add 20 μL of the 10 μM internal standard working solution, and 970 μL of the lipid reconstitution solution. Vortex mixture for 1 min, followed by centrifugation at 12,000 rpm and 4 °C for 10 min. Collect 120 μL of the resulting supernatant for LC-MS/MS analysis.

#### 2.3.3. Volatile Organic Compounds Preparation and Extraction

SO and RO samples were collected and stored at −80 °C until needed. Before processing, the samples were removed and placed on ice to thaw. One milliliter of sample was promptly transferred into a 20 mL headspace vial (Agilent, Palo Alto, CA, USA) preloaded with 0.2 g of NaCl powder to inhibit enzymatic activity. The vials were sealed with crimp-top caps fitted with TFE-silicone septa (Agilent). Prior to SPME analysis, each vial was incubated at 60 °C for 5 min, followed by exposure of a 120 µm DVB/CWR/PDMS SPME Arrow (Agilent) to the sample headspace for 15 min at the same temperature.

### 2.4. Instrument Conditions

#### 2.4.1. Chromatographic Separation Conditions

(1) UPLC conditions for widely targeted metabolites

UPLC conditions for widely targeted metabolites were consistent with those in our previous research [24]. A widely targeted metabolomic analysis was performed using a UPLC-ESI-MS/MS system (Shimadzu Nexera X2 UPLC, Kyoto, Japan; AB Sciex 4500 Q TRAP, Concord, ON, Canada). Chromatographic separation was carried out on an Agilent SB-C_18_ column (100 × 2.1 mm, 1.8 μm), employing 0.1% formic acid in water (solvent A) and acetonitrile (solvent B) as the mobile phases. The gradient elution program was as follows: 0–9 min, 95% to 5% solvent A; 9–10 min, 5% solvent A; 10–11.1 min, return to 95% solvent A; followed by equilibration until 14 min. The flow rate was 0.35 mL/min, the column temperature was maintained at 40 °C, and the injection volume was 4 μL.

(2) UPLC conditions for lipid compounds

The edible oil sample extracts were analyzed employing an LC-ESI-MS/MS system (UPLC, ExionLC AD; MS, QTRAP^®^ 6500+ System). The analytical column was a Thermo Accucore™ C30 (2.1 mm × 100 mm, 2.6 μm). The mobile phase consisted of solvent A (acetonitrile/water, 60/40 *v*/*v*, with 0.1% formic acid and 10 mmol/L ammonium formate) and solvent B (acetonitrile/isopropanol, 10/90 *v*/*v*, with 0.1% formic acid and 10 mmol/L ammonium formate). The gradient program was as follows: 80% A and 20% B at 0 min; a gradual shift from 0 to 2.0 min to 70% A and 30% B; from 2.0 to 4.0 min to 40% A and 60% B; from 4.0 to 9.0 min to 15% A and 85% B; from 9.0 to 14.0 min to 10% A and 90% B; from 14.0 to 15.5 min to 5% A and 95% B, maintained for 1.8 min; and from 17.3 to 20.0 min returning to 80% A and 20% B. The flow rate was set at 0.35 mL/min, the column temperature was maintained at 45 °C, and the injection volume was 2 μL.

(3) GC conditions for volatile organic compounds

Following sample collection, the volatile organic compounds (VOCs) were desorbed from the SPME Arrow coating by thermal desorption in the injection port of the GC system (Agilent Model 8890, Palo Alto, CA, USA) at 250 °C for 5 min, using a capillary column measuring 30 m × 0.25 mm × 0.25 μm (DB-5MS, 5% phenyl -polymethylsiloxane). Helium was used as the carrier gas, delivered at a constant linear flow rate of 1.2 mL/min. The injector temperature was maintained at 250 °C. The oven temperature was initially held at 40 °C for 3.5 min, then increased at 10 °C/min to 100 °C, followed by a ramp of 7 °C/min to 180 °C, then at 25 °C/min to 280 °C, where it was held for 5 min.

#### 2.4.2. MS/MS Conditions

(1) ESI-Q TRAP-MS/MS parameters

ESI-Q TRAP-MS/MS parameters were consistent with those used in our previous research [24]. Mass spectrometric detection was carried out on an AB Sciex 4500 Q TRAP system (Concord, ON, Canada), equipped with a Turbo Ion-Spray source and operated in both positive and negative ionization modes using Analyst 1.6.3 software. The source temperature was maintained at 550 °C, with ion spray voltages set at 5500 V and −4500 V for positive and negative modes, respectively. Nebulizer gas (Gas 1), heater gas (Gas 2), and Curtain Gas were set to 50, 60, and 25 psi, respectively. Instrument calibration and tuning were conducted using polypropylene glycol solutions (10 μmol/L for QQQ mode and 100 μmol/L for LIT mode). Data acquisition was conducted in multiple reaction monitoring (MRM) mode, using nitrogen as the collision gas at medium pressure.

(2) ESI-MS/MS parameters

Linear ion trap (LIT) and triple quadrupole (QQQ) mass spectra were acquired using a QTRAP^®^ 6500+ LC-MS/MS system (Concord, ON, Canada), a hybrid mass spectrometer combining triple quadrupole-linear ion trap technologies. The system was equipped with an ESI Turbo Ion-Spray source, operated in both positive and negative ionization modes, and controlled via Analyst 1.6.3 software (Sciex). The ESI source parameters were set as follows: ion source type, turbo spray; source temperature, 500 °C; ion spray voltage (IS), 5500 V in positive mode and −4500 V in negative mode; and gas pressures for Ion Source Gas 1 (GS1), Gas 2 (GS2), and Curtain Gas (CUR) were 45, 55, and 35 psi, respectively. Mass calibration and instrument tuning were carried out using polypropylene glycol solutions at concentrations of 10 μmol/L for QQQ mode and 100 μmol/L for LIT mode. Multiple reaction monitoring (MRM) experiments were performed in QQQ scan mode using nitrogen as the collision gas at a pressure of 5 psi. The declustering potential (DP) and collision energy (CE) for each MRM transition were optimized individually. A specific set of MRM transitions was monitored during each time segment, corresponding to the metabolites eluting within that interval.

(3) MS parameters

VOCs were identified and quantified using an Agilent 7000E mass spectrometer (Palo Alto, CA, USA). Mass spectra were acquired in electron ionization (EI) mode at an energy of 70 eV. The temperatures of the quadrupole mass analyzer, ion source, and transfer line were maintained at 150 °C, 230 °C, and 280 °C, respectively. The mass spectrometer was operated in selected ion monitoring (SIM) mode to facilitate the identification and quantification of target analytes.

### 2.5. Data Analysis of Metabolites

Data analysis of metabolites is consistent with our previous research [24]. Compound identification was performed by matching MS/MS spectra against the in-house library (MWDB) of Metware Biotechnology Co., Ltd. (Wuhan, China). During data processing, isotopic peaks and adduct ions (e.g., [M + K]^+^, [M + Na]^+^, [M + NH_4_]^+^) were filtered out, along with duplicate fragment ions originating from high-molecular-weight analogs.

Targeted metabolite quantification was performed using multiple reaction monitoring (MRM) mode on a triple quadrupole mass spectrometer. Precursor ions specific to each analyte were isolated in the first quadrupole (Q1), while non-target ions were excluded. These selected precursor ions were then fragmented in the collision cell (Q2) using argon gas, producing characteristic product ions that were further filtered by the third quadrupole (Q3) to ensure specificity. Raw mass spectrometry data were processed by integrating chromatographic peak areas, followed by manual correction to guarantee accurate metabolite quantification.

Metabolite identification was based on four key parameters: exact mass, MS/MS fragment ions, isotopic patterns, and retention time (RT). Spectral matching was performed against the MWDB database using Metware Biotechnology’s proprietary algorithm. The mass tolerance for precursor and fragment ions was set at 20 ppm, and the acceptable RT deviation was ≤0.2 min. Metabolites were assigned to three identification confidence levels: (1) High confidence: MS/MS spectral similarity ≥ 0.7. (2) Medium confidence: MS/MS spectral similarity between 0.5 and 0.7. (3) Putative identification: consistent precursor ion (Q1), product ion (Q3), and RT with reference standards in MWDB.

Multivariate statistical analyses, including principal component analysis (PCA), hierarchical cluster analysis (HCA), and orthogonal partial least squares discriminant analysis (OPLS-DA), were performed using R. Unsupervised PCA and HCA were performed after unit variance scaling of the dataset. Prior to OPLS-DA, the data were log_2_-transformed and mean-centered. Variable importance in projection (VIP) scores were extracted from the first predictive component of the OPLS-DA model, and model visualization was carried out using the MetaboAnalyst R package. To evaluate model robustness and prevent overfitting, a permutation test with 200 iterations was conducted. Differentially abundant metabolites were defined based on VIP ≥ 1 and |log_2_(fold change)| ≥ 1. Metabolites with significantly higher levels in Group A compared to Group B were considered upregulated, whereas those with lower levels were designated as downregulated.

Functional annotation of differentially abundant metabolites was performed by mapping them to the Kyoto Encyclopedia of Genes and Genomes (KEGG) database. Annotated metabolites were then assigned to their corresponding KEGG pathways. To identify biologically relevant mechanisms, metabolite set enrichment analysis (MSEA) was conducted on pathways containing significantly altered metabolites. Pathway enrichment significance was evaluated using the hypergeometric test, with a threshold of *p* < 0.05 considered statistically significant.

## 3. Results

### 3.1. Morphology of SO and RO

**Figure 1:**
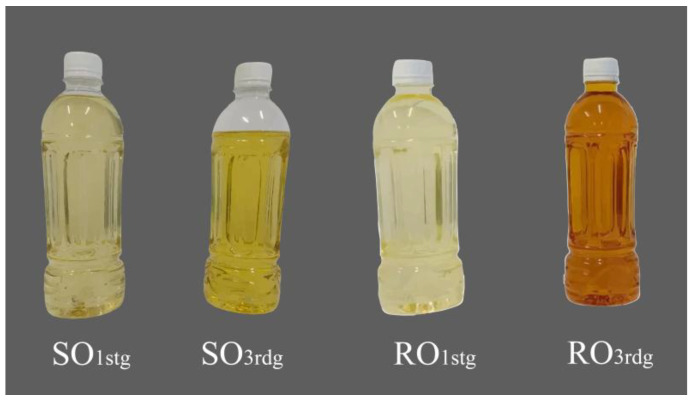
The appearances of SO and RO. SO_1stg_: First-grade soybean oil; SO_3rdg_: Third-grade soybean oil; RO_1stg_: First-grade rapeseed oil; RO_3rdg_: Third-grade rapeseed oil.

We selected SO (Figure 1: SO_1stg_ and SO_3rdg_) and RO (Figure 1: RO_1stg_ and RO_3rdg_) with different processing intensities as the research subjects. The color of these edible oils changes with the degree of processing intensities. The deeper the processing is, the lighter the color is. Overall, RO exhibits a darker color than SO. Notably, the color of RO_3rdg_ is significantly darker than that of SO_3rdg_ (Figure 1). These color differences may be attributed to variations in their chemical compositions.

### 3.2. Metabolic Profiling of SO and RO

Based on the local metabolite database, a total of 1033 widely targeted metabolites, 349 lipid compounds, and 157 volatile organic metabolites were identified through qualitative and quantitative analysis according to ion pair information from SO and RO samples (Table 1). The widely targeted metabolites included 95 alkaloids, 55 amino acids and derivatives, 80 flavonoids, 45 lignans and coumarins, 267 lipids, 9 nucleotides and derivatives, 40 organic acids, 57 phenolic acids, 7 quinones, 4 steroids, 201 terpenoids, and 173 other metabolites. The lipid compounds included 13 fatty acids (FA), 294 glycerolipids (GL), 31 glycerophospholipids (GP), 2 prenol lipids (PR), and 9 sphingolipids (SP). The volatile organic metabolites included 8 acids, 22 alcohols, 12 aldehydes, 8 amines, 6 aromatics, 22 esters, 2 halogenated hydrocarbons, 18 heterocyclic compounds, 20 hydrocarbons, 27 ketones, 2 nitrogen compounds, 1 phenol, 1 sulfur compound, and 8 terpenoids (Table 1).

### 3.3. Multivariate Analysis Revealed Differences Among the Metabolite Profiles

**Figure 2:**
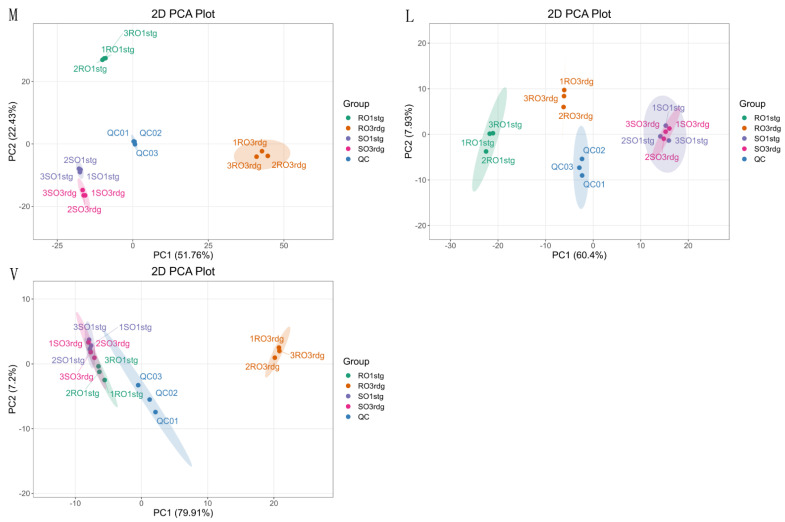
The plots of metabolomics (M), lipidomics (L), and volatileomics (V) illustrating the distribution of different treatments in vegetable oil examples. The PC1 and 2 demonstrate strong cohesion within groups and effective separation among the vegetable oil accessions.

Multivariate statistical analyses were conducted to evaluate variations in metabolic profiles across different treatment groups in SO and RO. Quality control (QC) samples, prepared by combining extracts from SO and RO, clustered closely within the same region, suggesting comparable metabolic profiles and demonstrating the stability and reproducibility of the analytical data (Figure 2). However, these edible oil samples exhibited distinct clustering patterns in the heatmap (Figure S1). In the PCA plot, the first two principal components of widely targeted metabolites (PC1 and PC2) explained 51.76% and 22.43% of the data variance, respectively (Figure 2 M). The 15 samples, derived from five treatments across two types of edible oils, clustered into five separate groups, indicating that each group of widely targeted metabolites exhibited a unique metabolic profile (Figure 2 M). For lipid compounds, the first two principal components (PC1 and PC2) explained 60.4% and 7.93% of the data variance, respectively (Figure 2 L). The 15 samples, representing five treatments across two edible oil types, were classified into four distinct clusters, indicating that each group of lipid compounds displayed a unique metabolic profile (Figure 2 L). SO_1stg_ and SO_3rdg_ suggest that processing has a relatively minor impact on the lipid composition of SO, as the lipid profiles of SO_1stg_ and SO_3stg_ were similar (Figure 2 L). Regarding volatile organic compounds, the first two principal components (PC1 and PC2) explained 79.91% and 7.2% of the data variance, respectively (Figure 2 V). The 15 samples, derived from five treatments across two edible oil types, clustered into three distinct groups, indicating that each group of volatile organic compounds exhibited a unique metabolic profile (Figure 2 V). The grouping of SO_1stg_, SO_3rdg_, and RO_1stg_ indicates that processing has a relatively minor impact on the volatile composition of SO, as the volatile profiles of SO_1stg_ were similar to those of SO_3rdg_ and RO_1stg_ (Figure 2 V). Overall, the PCA results demonstrated that different treatments can be distinguished from each other, indicating that both variety and processing intensity significantly impact the metabolism of edible oils. The effect of variety on metabolic profiles appeared to be greater than that of processing (Figure 2).

**Figure 3:**
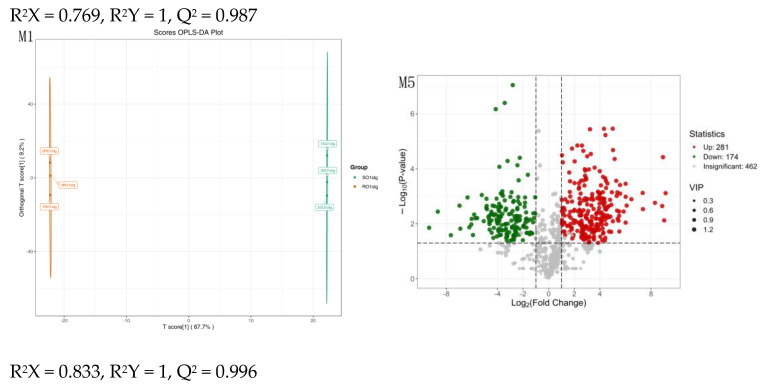

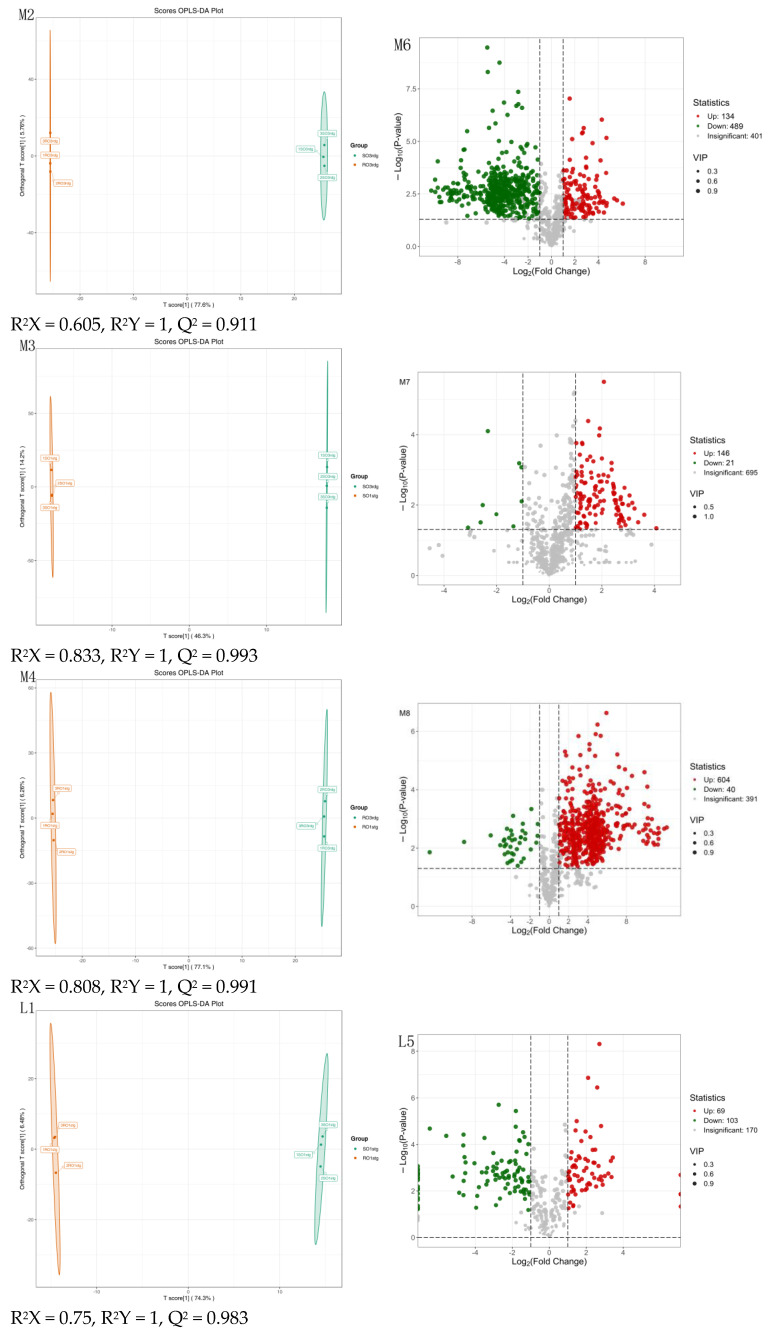

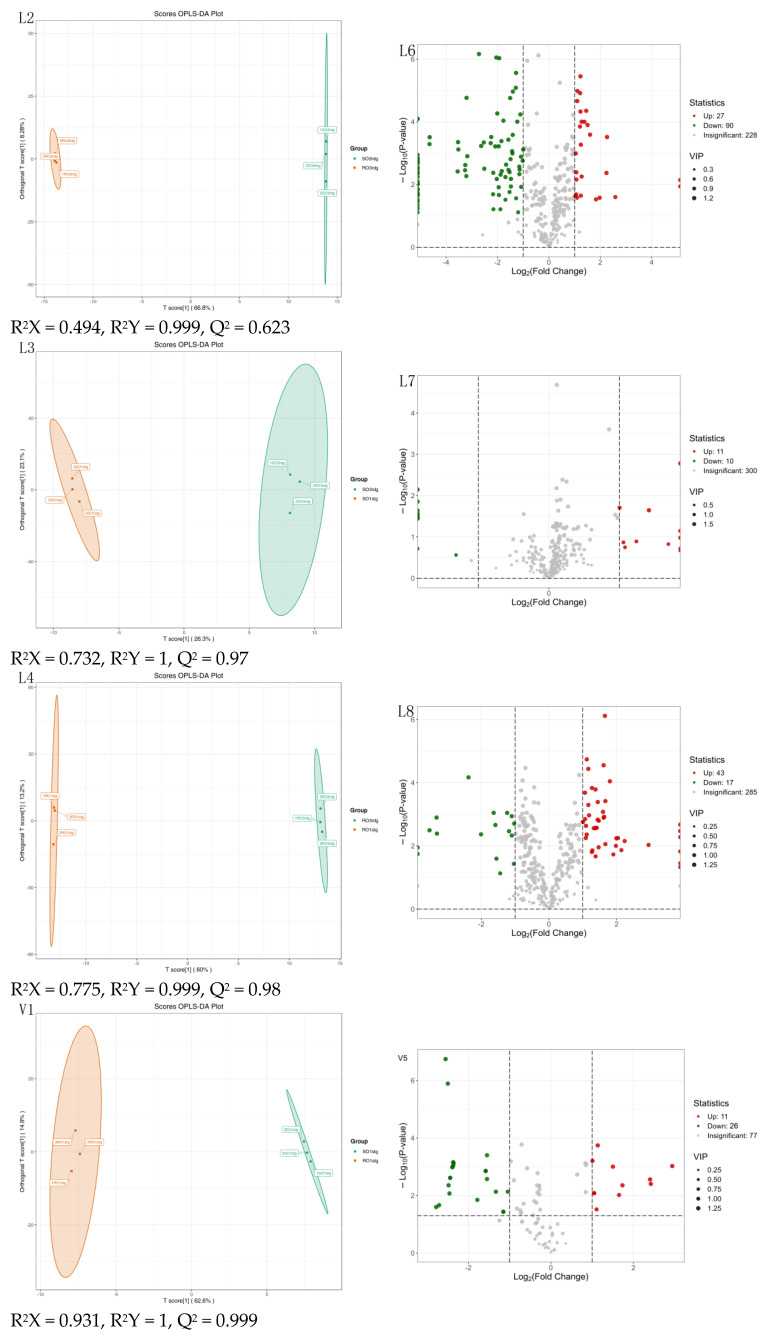

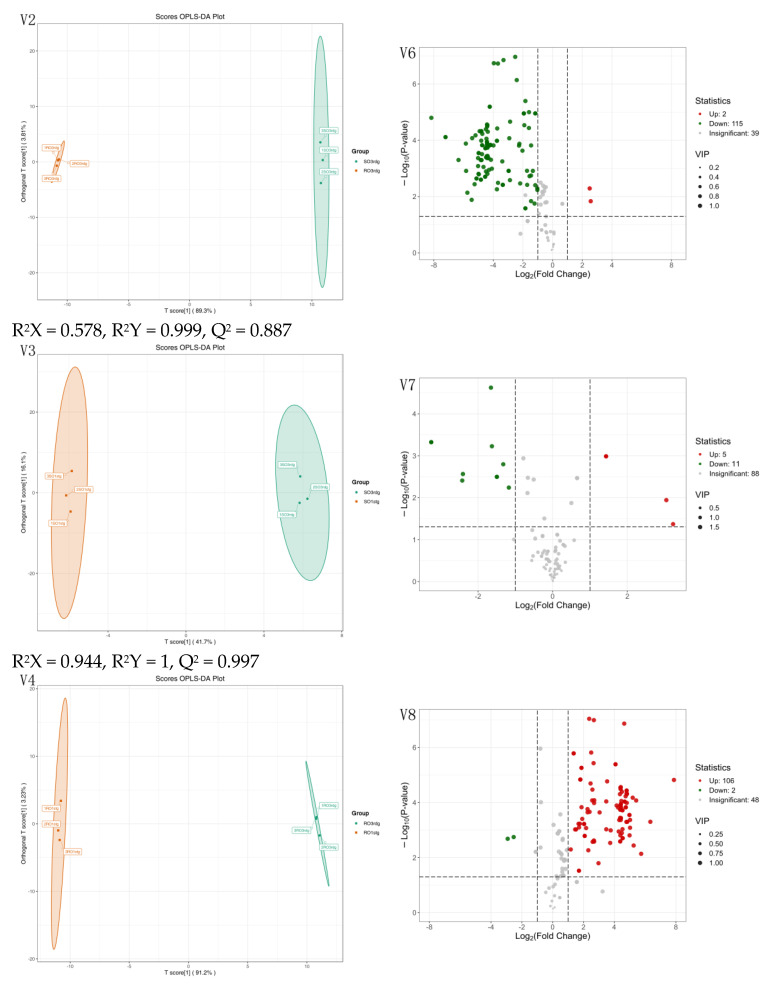
The OPLS-DA score chart displays the comparison of 4 groups of vegetable oil, namely SO_1stg_ vs. RO_1stg_, SO_3rdg_ vs. RO_3rdg_, SO_3rdg_ vs. SO_1stg,_ and RO_3rdg_ vs. RO_1stg_. M: metabolomics; L: lipidomics; V: volatileomics.

OPLS-DA was employed to identify the variables responsible for the variations observed among the four groups of widely targeted metabolites. In this study, the OPLS-DA model was applied to assess the metabolic differences between SO_1stg_ and RO_1stg_ (R^2^X = 0.769, R^2^Y = 1, Q^2^ = 0.987), between SO_3rdg_ and RO_3rdg_ (R^2^X = 0.833, R^2^Y = 1, Q^2^ = 0.996), between SO_3rdg_ and SO_1stg_ (R^2^X = 0.605, R^2^Y = 1, Q^2^ = 0.911), between RO_3rdg_ and RO_1stg_ (R^2^X = 0.833, R^2^Y = 1, Q^2^ = 0.993) (Figure 3 M1–M4). OPLS-DA was also used to identify the variables responsible for variations among the four groups of lipid compounds. The model assessed metabolic differences between SO_1stg_ and RO_1stg_ (R^2^X = 0.808, R^2^Y = 1, Q^2^ = 0.991), between SO_3rdg_ and RO_3rdg_ (R^2^X = 0.75, R^2^Y = 1, Q^2^ = 0.983), between SO_3rdg_ and SO_1stg_ (R^2^X = 0.494, R^2^Y = 0.999, Q^2^ = 0.623), between RO_3rdg_ and RO_1stg_ (R^2^X = 0.732, R^2^Y = 1, Q^2^ = 0.97) (Figure 3 L1–L4). Furthermore, OPLS-DA was applied to identify variables responsible for variations among the four groups of volatile organic compounds. The model evaluated metabolic differences between SO_1stg_ and RO_1stg_ (R^2^X = 0.775, R^2^Y = 0.999, Q^2^ = 0.98), between SO_3rdg_ and RO_3rdg_ (R^2^X = 0.931, R^2^Y = 1, Q^2^ = 0.999), between SO_3rdg_ and SO_1stg_ (R^2^X = 0.578, R^2^Y = 0.999, Q^2^ = 0.887), between RO_3rdg_ and RO_1stg_ (R^2^X = 0.944, R^2^Y = 1, Q^2^ = 0.997) (Figure 3 V1–V4).

Pairwise analyses were performed across various edible oil treatments to identify the metabolites responsible for the observed variations. The results demonstrated a clear separation among the different treatment groups. Using the criteria of FC ≥ 2 or ≤0.5 and VIP ≥ 1, there were 455 differential widely targeted metabolites between SO_1stg_ and RO_1stg_ (281 upregulated, 174 downregulated), 623 between SO_3rdg_ and RO_3rdg_ (134 upregulated, 489 downregulated), 167 between SO_3rdg_ and SO_1stg_ (146 upregulated, 21 downregulated), and 644 between RO_3rdg_ and RO_1stg_ (604 upregulated, 40 downregulated) (Figure 3 M5–M8). There were 172 differential lipid metabolites between SO_1stg_ and RO_1stg_ (69 upregulated, 103 downregulated), 117 between SO_3rdg_ and RO_3rdg_ (27 upregulated, 90 downregulated), 21 between SO_3rdg_ and SO_1stg_ (11 upregulated, 10 downregulated), and 60 between RO_3rdg_ and RO_1stg_ (43 upregulated, 17 downregulated) (Figure 3 L5–L8). There were 37 differential volatile organic metabolites between SO_1stg_ and RO_1stg_ (11 upregulated, 26 downregulated), 117 between SO_3rdg_ and RO_3rdg_ (2 upregulated, 115 downregulated), 16 between SO_3rdg_ and SO_1stg_ (5 upregulated, 11 downregulated), and 108 between RO_3rdg_ and RO_1stg_ (106 upregulated, 2 downregulated) (Figure 3 V5–V8). The differences in metabolite subtypes among the omics datasets are detailed in Table S1.

**Figure 4:**
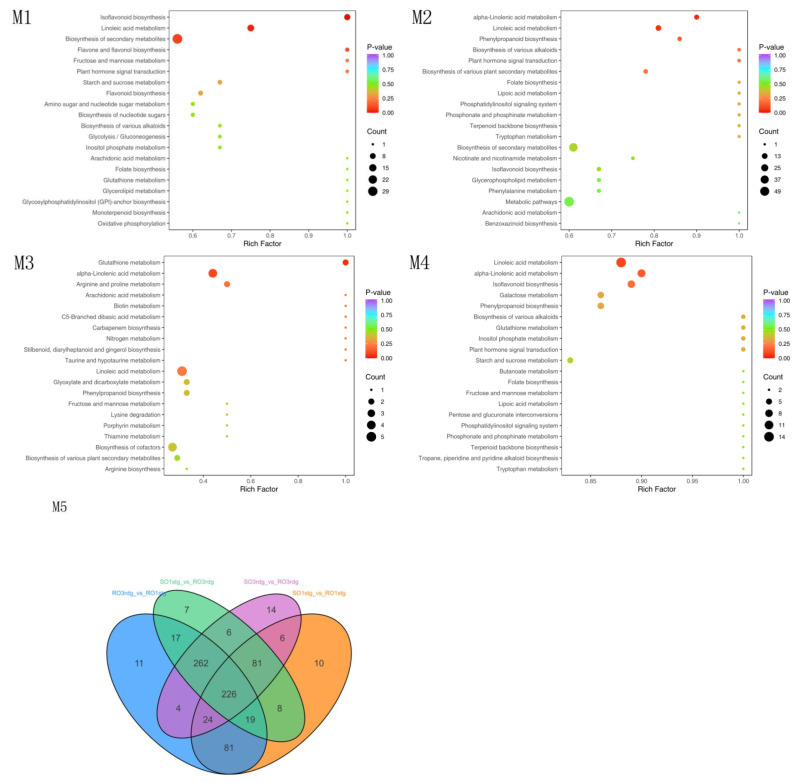

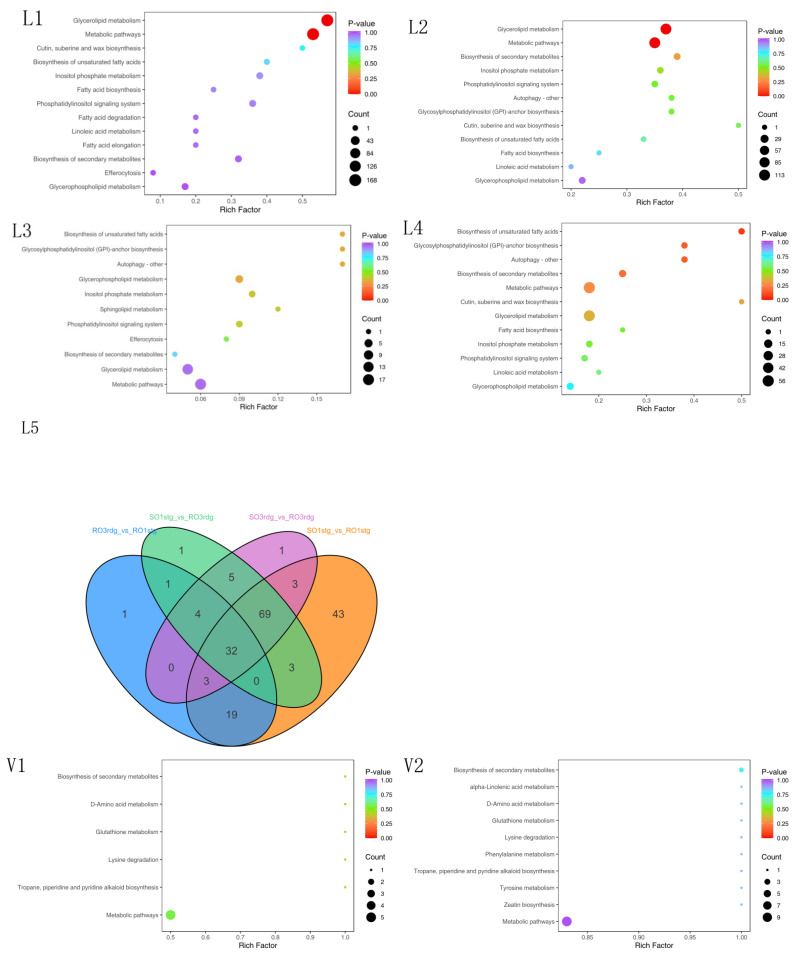

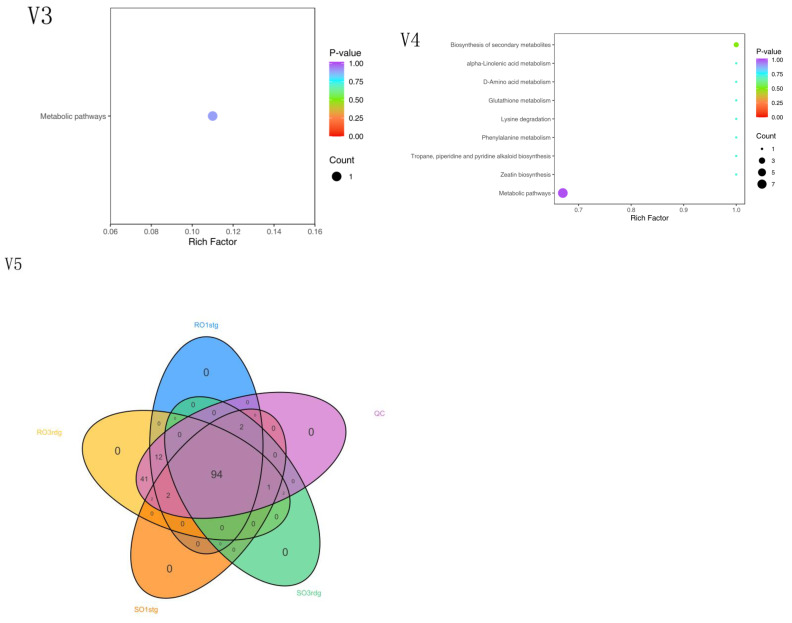
Visual representations including Venn diagrams and bubble diagrams were utilized to illustrate comparisons among three distinct sets of differentially expressed metabolites (SO_1stg_ vs. RO_1stg_, SO_3rdg_ vs. RO_3rdg_, SO_3rdg_ vs. SO_1stg_ and RO_3rdg_ vs. RO_1stg_). M: metabolomics; L: lipidomics; V: volatileomics. In Figure M5, L5, V5, a Venn diagram was employed to showcase both shared and unique metabolites across these comparison groups. Additionally, Figures M1–M4, L1–L4 and V1–V4 present KEGG enrichment analyses for differentially expressed metabolites within each group. Each individual bubble within these figures corresponds to a specific metabolic pathway; its position on an axis as well as its size collectively indicate its level of influence within that particular pathway. Larger bubbles signify greater influence factors. Furthermore, variations in bubble color reflect *p* values obtained from enrichment analysis with darker colors indicating higher degrees of enrichment.

The KEGG database facilitates the analysis of metabolite accumulation within a comprehensive metabolic network. In this study, differential metabolites from each comparison group were functionally enriched and categorized into distinct metabolic pathways. In the comparison between SO_1stg_ and RO_1stg_, the widely targeted metabolites were significantly enriched in metabolic pathways associated with “isoflavonoid biosynthesis”, “linoleic acid metabolism”, “biosynthesis of secondary metabolites”, and “flavone and flavonol biosynthesis” (*p* < 0.05) (Figure 4 M1). In the comparison between SO_3rdg_ and RO_3rdg_, significantly enriched metabolic pathways included “alpha-linolenic acid metabolism”, “linoleic acid metabolism” (*p* < 0.05) (Figure 4 M2). The comparison of SO_3rdg_ and SO_1stg_ revealed significant enrichment in “glutathione metabolism”, “alpha-linolenic acid metabolism” and “linoleic acid metabolism” (*p* < 0.05) (Figure 4 M3). Similarly, the comparison of RO_3rdg_ and RO_1stg_ showed significant enrichment in “linoleic acid metabolism”, “alpha-linolenic acid metabolism” and “isoflavonoid biosynthesis” (*p* < 0.05) (Figure 4 M4). Venn diagram analysis indicated that 226 metabolites were common across all four comparison groups (Figure 4 M5). Regarding lipid compounds, the comparison between SO_1stg_ and RO_1stg_ revealed significant enrichment in “glycerolipid metabolism” and “metabolic pathways” (*p* < 0.05) (Figure 4 L1). Similarly, “glycerolipid metabolism” and “metabolic pathways” were significantly enriched in the comparison between SO_3rdg_ and RO_3rdg_ (Figure 4 L2). No significantly enriched metabolic pathways were identified in the comparison of SO_3rdg_ and SO_1stg_ (Figure 4 L3). In the comparison of RO_3rdg_ and RO_1stg_, significant enrichment was observed in “biosynthesis of unsaturated fatty acids”, “glycosylphosphatidylinositol (GPI)-anchor biosynthesis” “autophagy-other” and “biosynthesis of secondary metabolites” (*p* < 0.05) (Figure 4 L4). Venn diagram analysis showed that 32 lipid compounds were common across the four comparison groups (Figure 4 L5). No significantly enriched metabolic pathways were found among volatile organic compounds across different treatments (Figure 4 V1–V4). However, Venn diagram analysis showed that 94 volatile organic compounds were common across all four comparison groups (Figure 4 V5). These findings indicate that the metabolites responsible for the observed differences exhibited significant variation.

### 3.4. Mining and Analysis of Characteristic Metabolites

**Figure 5:**
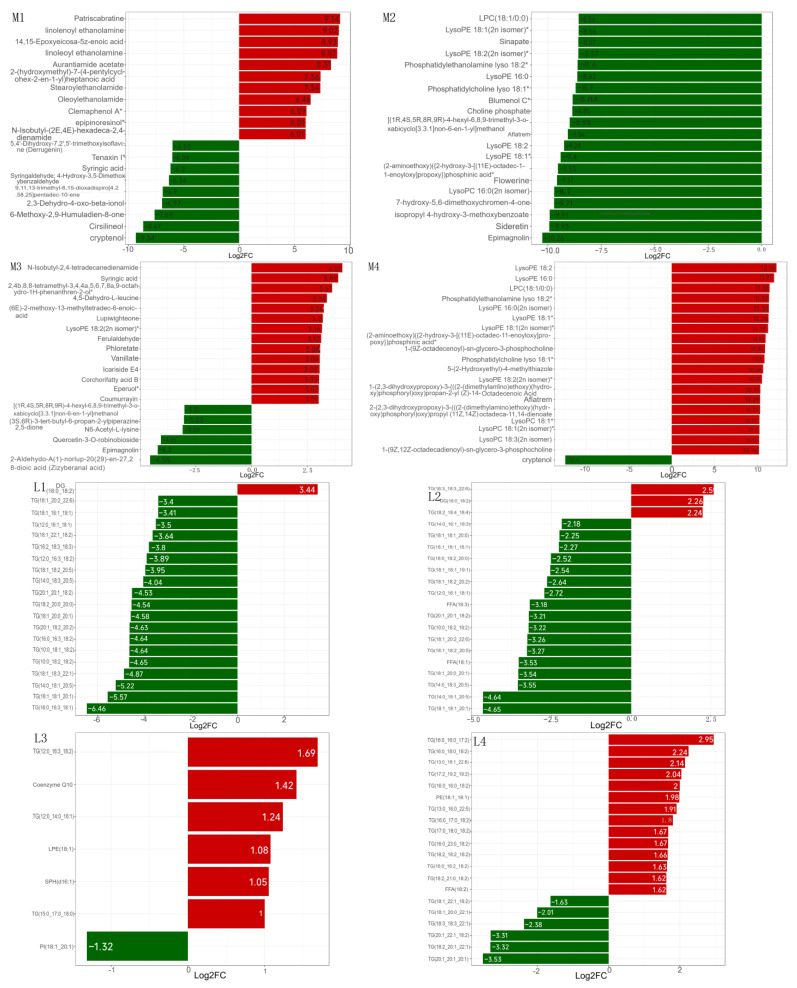

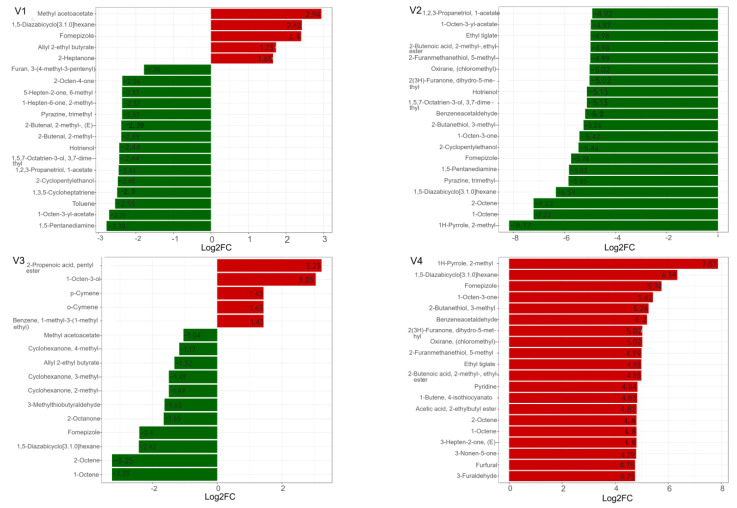
The highest fold change was observed in the top 7~20 metabolites that were upregulated and downregulated in SO_1stg_ vs. RO_1stg_, SO_3rdg_ vs. RO_3rdg_, SO_3rdg_ vs. SO_1stg_ and RO_3rdg_ vs. RO_1stg_ comparison groups. M: metabolomics; L: lipidomics; V: volatileomics. * indicates isomers. The upregulated metabolites are represented by red bar charts while the downregulated metabolites are represented by green bar charts.

To further investigate the characteristic metabolites in each comparison group, the top seven to 20 differential metabolites exhibiting the highest FC were selected from each group (Figure 5). Among these, the 11 upregulated widely targeted metabolites in the SO_1stg_ vs. RO_1stg_ comparison (Figure 5 M1) included four alkaloids, five lipids, and two lignans and coumarins. The nine downregulated widely targeted metabolites were three flavonoids, one phenolic acid, three terpenoids, one lipid, and one other metabolite. In the comparison of SO_3rdg_ vs RO_3rdg_ (Figure 5 M2), the top 20 differential widely targeted metabolites with the highest FC were all downregulated, including ten lipids, two phenolic acids, two terpenoids, two alkaloids, two lignans and coumarins, one flavonoid, and one other metabolite. In the comparison of SO_3rdg_ vs SO_1stg_ (Figure 5 M3), 14 widely targeted metabolites were upregulated, comprising one alkaloid, three phenolic acids, two terpenoids, one amino acid and derivative, three lipids, one flavonoid, two lignans and coumarins, and one other metabolite. While six were downregulated, including two terpenoids, one alkaloid, one amino acid and derivative, one flavonoid, and one lignan and coumarin. In the comparison of RO_3rdg_ vs RO_1stg_ (Figure 5 M4), 17 lipids, one alkaloid, and one other metabolite were regulated, while the one lipid was downregulated.

The 20 lipid compounds with the highest FC were selected as the most significantly differentially expressed in each comparison group (Figure 5). Among these, one upregulated lipid compound was GL, and 19 downregulated lipid compounds were GL in the comparison of SO_1stg_ vs. RO_1stg_ (Figure 5 L1). In the comparison of SO_3rdg_ vs. RO_3rdg_ (Figure 5 L2), three lipid compounds GLs were upregulated, while 17 lipid compounds were downregulated including 15 GLs and two FAs. In the comparison of SO_3rdg_ vs. SO_1stg_ (Figure 5 L3), six upregulated lipid compounds were upregulated, including three GLs, one PR, one GP, and one SP, while lipid compound GP was downregulated. In the comparison of RO_3rdg_ vs. RO_1stg_ (Figure 5 L4), 14 lipid compounds were upregulated including 12 GLs, one GP, and one FA, while the six lipid compounds GLs were downregulated.

The five upregulated volatile organic compounds included two esters, two heterocyclic compounds, and one ketone, while the 15 downregulated volatile organic compounds comprised one terpenoid, three ketones, one heterocyclic compound, two aldehydes, three alcohols, two esters, one hydrocarbon, one aromatic, and one amine in the comparison between SO_1stg_ and RO_1stg_ (Figure 5 V1). The top 20 differential volatile organic compounds with the highest FC were all downregulated in the comparison between SO_3rdg_ and RO_3rdg_. They included four esters, five alcohols, one halogenated hydrocarbon, two ketones, one aldehyde, four heterocyclic compounds, one amine, and two hydrocarbons (Figure 5 V2). The five upregulated volatile organic compounds in the comparison between SO_3rdg_ and SO_1stg_ included one ester, one alcohol, and three terpenoids, while the 11 downregulated compounds consisted of two esters, four ketones, one aldehyde, two heterocyclic compounds, and two hydrocarbons (Figure 5 V3). The top 20 differential volatile organic compounds with the highest FC were all upregulated in the comparison of RO_3rdg_ and RO_1stg_. These included two heterocyclic compounds, six ketones, three alcohols, three aldehydes, one halogenated hydrocarbon, three esters, and two hydrocarbons (Figure 5 V4). These compounds can be selectively used as biomarkers for detecting adulteration in edible oils.

Interestingly, these compounds of TG (16:0_18:2_22:2), TG (20:1_22:1_18:2), TG (18:1_20:2_22:1), TG (18:2_20:1_22:1), TG (12:0_18:1_22:6), TG (18:3_18:3_22:1), TG (24:1_18:3_18:3), TG (24:1_18:2_18:3), TG (18:2_16:3_18:3), TG (12:0_16:3_18:3), TG (12:0_18:3_18:3), TG (17:1_18:1_20:1), TG (18:1_20:2_20:0), TG (18:0_20:1_20:2), TG (20:1_20:1_20:1), TG (18:1_20:0_22:1), and TG (16:0_18:1_24:1) are present in the RO_3rdg_ group. After further processing and transformation into the RO_1stg_ group, these compounds remain detectable (Table S2). However, none of these substances were detected in either the SO_3rdg_ or SO_1stg_ groups. Compounds found exclusively in the SO_3rdg_ group include TG (15:0_17:0_18:0), and Coenzyme Q9. Conversely, compounds unique to the RO3rdg group are TG (16:0_16:3_18:2), TG (13:0_16:3_18:2), TG (16:2_18:3_18:3), TG (18:3_20:1_20:2), TG (20:1_18:2_20:2), TG (18:1_18:3_22:1), TG (18:1_20:2_20:2), TG (16:0_16:3_18:1), TG (18:0_18:2_22:1), TG (16:0_18:1_24:1), TG (18:2_20:0_20:0), TG (10:0_18:1_18:2), PE (18:2_18:2), and PE (18:1_18:2). Compounds found only in the SO_1stg_ group include TG (13:0_16:3_18:2), TG (16:0_17:1_20:4), TG (18:2_18:4_18:4), and TG (16:3_18:3_22:6). Meanwhile, compounds unique to the RO_1stg_ group are DG (18:1_18:3), TG (16:3_18:2_22:5), TG (14:0_16:1_18:3), TG (16:0_18:1_24:1), and FFA (15:1) (Table S2).

After further processing, the substances PE (18:2_18:2), PE (18:1_18:2), TG (13:0_16:3_18:2), TG (16:0_17:1_20:4), TG (18:2_18:4_18:4), and FFA (20:2) in the RO_1stg_ group were no longer detected (Table S2). Similarly, these substances FFA (20:2), DG (18:1_18:3), TG (16:3_18:2_22:5), TG (14:0_16:1_18:3), and LPC (14:0) in the SO_1stg_ group were also undetectable (Table S2). These results demonstrate that processing intensity significantly affects the compound composition of edible oils.

## 4. Discussion

### 4.1. Metabolites Identified of SO and RO

EPO is an important source of dietary fat and fat-soluble nutrients worldwide. Its safety and nutritional quality are directly related to consumer rights and public health. SO and RO have become the two most widely consumed vegetable oils globally due to their high yields and relatively balanced fatty acid compositions [2]. Therefore, SO and RO obtained through physical pressing were selected as the research subjects. The appearance showed that after pressing and refining into various grades of oil, their colors differed significantly, with both oils changing from dark to light (Figure 1). Widely targeted metabolomics, lipidomics, and volatileomics were employed to investigate the effects of variety and processing intensity on the compound composition of SO and ROs in the present study, providing a comprehensive metabolite profile of SO and RO. A total of 1033 widely targeted metabolites, 349 lipid compounds, and 157 volatile organic metabolites were identified in SO and RO samples (Table 1). The amounts and types of metabolites in SO and RO differed, consistent with previous studies [1]. Our results confirmed the first hypothesis proposed in this study, namely that RO and SO have distinct metabolomic profiles. With advances in detection technology, new compounds are continually being identified [25]. Currently, methods for studying the chemical composition of EPO comprise spectroscopic and chromatographic techniques. Spectroscopic methods include NIR, FTIR, UV-Vis, Raman and fluorescence spectroscopy, NMR and ICP-OES; chromatographic methods include GC, GC/MS, HPLC, HPLC/MS, etc. [17]. For example, researchers have used GC-MS to investigate fatty acids, squalene, and phytosterols in different EPO [16], while other studies have employed lipidomics to study camellia oil [26]. However, to our knowledge, relatively few studies have used multi-omics approaches to investigate the metabolites of EPO. Therefore, these results contribute to a better understanding of the chemical composition and quality differences among various EPOs.

### 4.2. The Impact of Processing on the Components of RO and SO

We conducted a multivariate analysis, which revealed that the processing procedure significantly reduces the relative content of certain compounds in EPO. Importantly, the upregulated differential metabolites (604 widely targeted metabolites, 43 lipid compounds, and 106 volatile organic compounds) in the comparison between RO_3rdg_ and RO_1stg_ accounted for 93.79%, 71.67%, and 98.15% of the total differential metabolites (644 widely targeted metabolites, 60 lipid compounds, and 108 volatile organic compounds), respectively. Similarly, the upregulated differential metabolites (146 widely targeted metabolites, 11 lipid compounds, and five volatile organic compounds) in the comparison between SO_3rdg_ and SO_1stg_ accounted for 87.43%, 52.38%, and 31.25% of the total differential metabolites (167 widely targeted metabolites, 21 lipid compounds, and 16 volatile organic compounds), respectively. These findings align with previous studies [8], which demonstrated that the refining process destroys micronutrients, resulting in the losses of 10–36% of total tocopherols, 6–52% of total sterols, and 93–98% of polyphenols [8]. Other research has shown that deodorization significantly affects the phytosterols and vitamin E content in SO, while its impact on RO’s trace elements is minor, with losses exceeding 10% [5]. Furthermore, six substances present in the RO_3rdg_ group were undetectable in the RO_1stg_ group, and similarly, five substances found in the SO_srdg_ group were absent in the SO_1stg_ (Table S2). Our multi-omics results also indicate that processing significantly affects the chemical composition of EPO, with affects varying according to the type of EPO. Therefore, processing parameters should be optimized to minimize nutrient loss during EPO production [5,8].

Metabolic pathways related to “isoflavonoid biosynthesis” were markedly enriched (*p* < 0.05) in the comparison between RO_3rdg_ and RO_1stg_ (Figure 4 M4). Notably, the relative content of 76.47% of flavonoids, 100% of organic acids, and 85% of phenolic acids in the RO_1stg_ group was significantly lower than that in the RO_3rdg_ group (Table S1). Flavonoids play important physiological roles in biomedical and health-related fields, functioning as antioxidants by scavenging free radicals, acting as reducing agents, and quenching singlet oxygen [27]. They are potent antioxidant compounds exhibiting a range of biological and pharmacological effects, including anti-inflammatory and anticancer activities [27]. For example, naringenin demonstrates anticancer, antioxidant, anti-inflammatory, and antiproliferative properties [28,29], while kaempferol-3-*O*- glucoside exhibits anti-inflammatory, antioxidant, and protective effects [30]. Phenolic acids possess hydrogen peroxide scavenging, antioxidant, and anti-radical activity [31], and organic acids also exhibit antioxidant and anti-radical activity [32]. To obtain more natural antioxidant substances through the consumption of EPO, it is recommended that consumers select an appropriate amount of SO_3rdg_ and RO_3rdg_. Furthermore, the relative content of 69.23% of GL in the RO_1stg_ group was significantly lower than that in the RO_3rdg_ group (Table S1). These findings indicate that metabolomics and lipidomics provide valuable data and a theoretical basis for the quality and safety control of EPO [33]. Therefore, these results have important theoretical and practical implications for guiding consumers in making informed choices regarding different processing grades of EPO, as well as for the processing of SO_3rdg_ and RO_3rdg_. Our study provides data support for the quality evaluation of EPO proposed by previous researchers [34,35]. We have confirmed the second hypothesis of this study, demonstrating that processing significantly impacts the metabolites of RO and SO.

### 4.3. Possible Biomarker of Authentication

The seven to 20 differential compounds with the highest FCs in each treatment comparison group were selected for further analysis (Figure 5). Based on significant changes in quantity, the main substances identified were GL, lipids, terpenoids, flavonoids, alkaloids, ketones, and esters in the SO_1stg_ vs. RO_1stg_ comparison group; lipids, terpenoids, GL, alkaloids, phenolic acids, alcohols, and heterocyclic compounds in the SO_3rdg_ vs. RO_3rdg_ comparison group; phenolic acids, lipids, GL, terpenoids and ketones in the SO_3rdg_ vs. SO_1stg_ comparison group; lipids, terpenoids, GL, ketones, and alcohols in the RO_3rdg_ vs. RO_1stg_ comparison group. These results are consistent with previous studies, which indicate that chemical markers for food authentication include volatiles, phenolic compounds, natural pigments, elements, isotopes, and more [17]. Therefore, these substances can be considered potential biomarker compounds for identifying the presence of SO impurities in RO, and vice versa.

Through further analysis of the data from the three omics approaches, the results indicate that lipidomics provides more valuable information regarding the adulteration of RO and SO. The TG (15:0_17:0_18:0), and coenzyme Q_9_ are present only in the SO_3rdg_ group. However, 12 compounds are found exclusively in the RO_3rdg_ group. Additionally, one compounds exist only in the SO_1stg_ group while one compounds are unique to the RO_1stg_ group (Table S2). Our findings are consistent with previous studies [16,18]. Previous research has shown that isoflavones and resveratrols can be detected in SO but not in peanut oil, making them effective biomarkers to distinguish SO from peanut oil in cases of adulteration and forgery [18]. Researchers have also identified stigmasterol, obtusifoliol, and cycolartenol in SO, but not in RO [16]. Interestingly, 17 TGs are present in the RO_3rdg_ group and persist after further processing in RO_1stg_; however, these substances were not detected in either the SO_3rdg_ or SO_1stg_ groups (Table S2). Refining not only reduces the quantity of compounds in edible oils, but also decreases their concentration [36]. Our results suggest that these substances can be used to identify adulteration in SO_3rdg_ and RO_3rdg_, as well as to detect fraud in SO_1stg_ and RO_1stg_ after refining. Future studies should increase the sample size with appropriate training and validation datasets to further validate these characteristic compounds. Additionally, integrating artificial intelligence technology could improve the efficiency and cost-effectiveness of this non-destructive method [37], providing data support and a theoretical basis for establishing regulatory system in the EPO and fat industry.

## 5. Conclusions

Our research evaluated the impact of varieties and processing methods on metabolites in EPO from a multi-omics perspective. SO and RO exhibited different compound profiles. Oil processing and refining significantly affected these compounds; therefore, processing methods should be optimized to minimize nutrient loss during production. Lipid substances can serve as biomarkers for mixtures of SO and RO. Since the varieties and sample sizes in this study were relatively small, further research should include a greater variety and larger sample sizes to improve the method’s intensity. Additionally, future studies should integrate artificial intelligence technologies to enhance the efficiency and reduce the cost of the method from a non-destructive perspective, providing data support and a theoretical basis for developing regulatory systems in the EPO and fat industries.

## Figures and Tables

**Table 1:** Overview of annotated metabolites.

Metabolic Type		Number	Percentage (%)
Widely targeted metabolites (1033)	Alkaloids	95	9.20
	Amino acids and derivatives	55	5.32
	Flavonoids	80	7.74
	Lignans and coumarins	45	4.36
	Lipids	267	25.85
	Nucleotides and derivatives	9	0.87
	Organic acids	40	3.87
	Phenolic acids	57	5.52
	Quinones	7	0.68
	Steroids	4	0.39
	Terpenoids	201	19.46
	Other metabolites	173	16.75
Lipid compounds (349)	Fatty acids (FA)	13	3.72
	Glycerolipids (GL)	294	84.24
	Glycerophospholipids (GP)	31	8.88
	Prenol lipids (PR)	2	0.57
	Sphingolipids (SP)	9	2.58
Volatile organic compounds (157)	Acids	8	5.10
	Alcohols	22	14.01
	Aldehydes	12	7.64
	Amines	8	5.10
	Aromatics	6	3.82
	Esters	22	14.01
	Halogenated hydrocarbons	2	1.27
	Heterocyclic compounds	18	11.46
	Hydrocarbons	20	12.74
	Ketones	27	17.20
	Nitrogen compounds	2	1.27
	Phenols	1	0.64
	Sulfur compounds	1	0.64
	Terpenoids	8	5.10

## Data Availability

The original contributions presented in this study are included in the article/Supplementary Materials. Further inquiries can be directed to the corresponding authors.

## Supplementary Materials

The following supporting information can be downloaded at https://www.mdpi.com/article/10.3390/foods15152738/s1, Figure S1. Heat maps of metabolomics (M), lipidomics (L) and volatileomics (V) illustrating the distribution of metabolites across different treatments in vegetable oil examples. Table S1. Metabolites significantly changed in different treatments. Table S2. Possile biomarkers of lipid compounds.
