# Supplementary material for: Multi-Omics Reveals the Effects of Processing on Chemical Composition of Different Vegetable Oils
Source: Foods. 2026 Aug 4;15(15):2738. doi: 10.3390/foods15152738 (PMC13465484; doi:10.3390/foods15152738)
Supplement: Supplementary file 1 [file foods-15-02738-s001.zip › Supplement Figure S1.pdf]

M

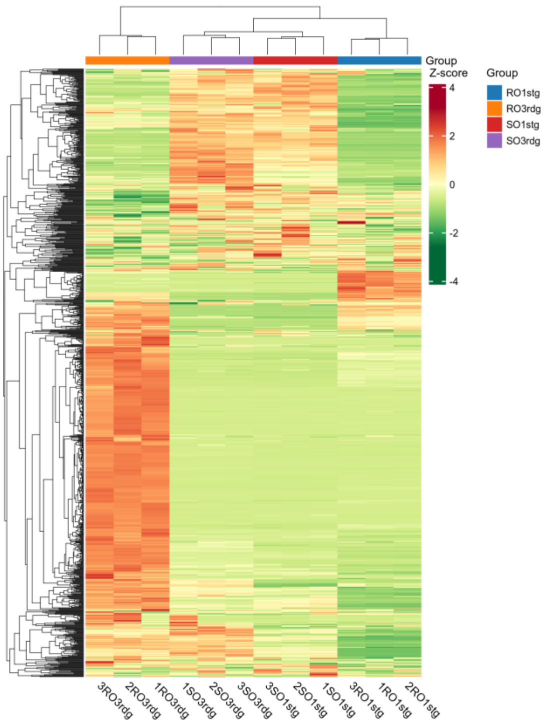
Group
Z-score
4
2
0
-2
-4
Group
RO1stg
RO3rdg
SO1stg
SO3rdg
3RO3rdg
2RO3rdg
1RO3rdg
1SO3rdg
2SO3rdg
3SO3rdg
3SO1stg
2SO1stg
1SO1stg
3RO1stg
1RO1stg
2RO1stg

L

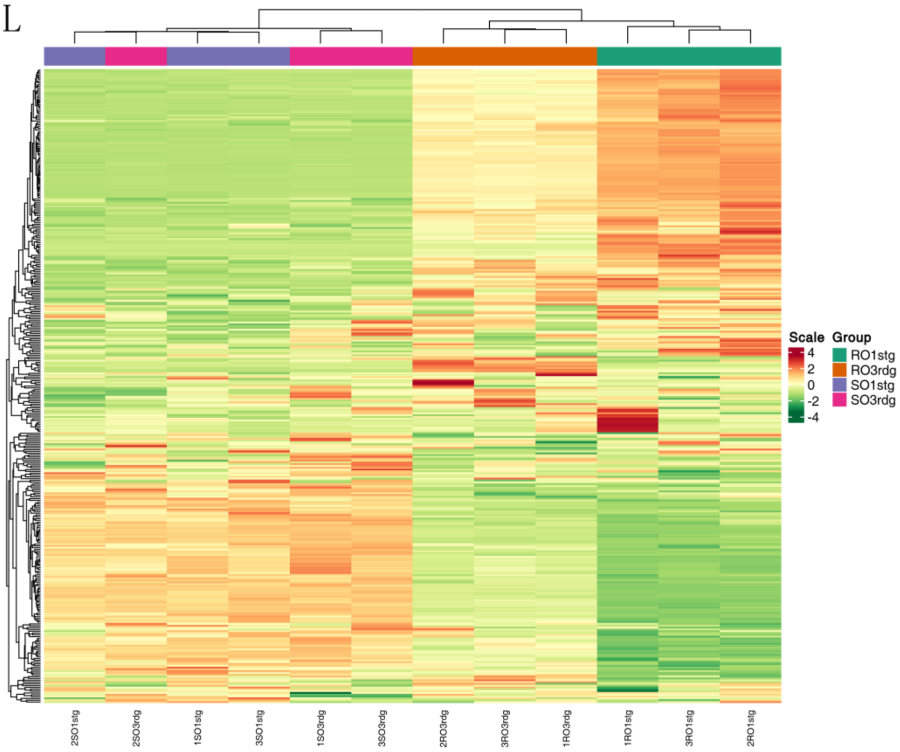
Scale
Group
4
2
0
-2
-4
RO1stg
RO3rdg
SO1stg
SO3rdg
2SO1stg
2SO3rdg
1SO1stg
3SO1stg
1SO3rdg
3SO3rdg
2RO3rdg
3RO3rdg
1RO3rdg
1RO1stg
3RO1stg
2RO1stg

V

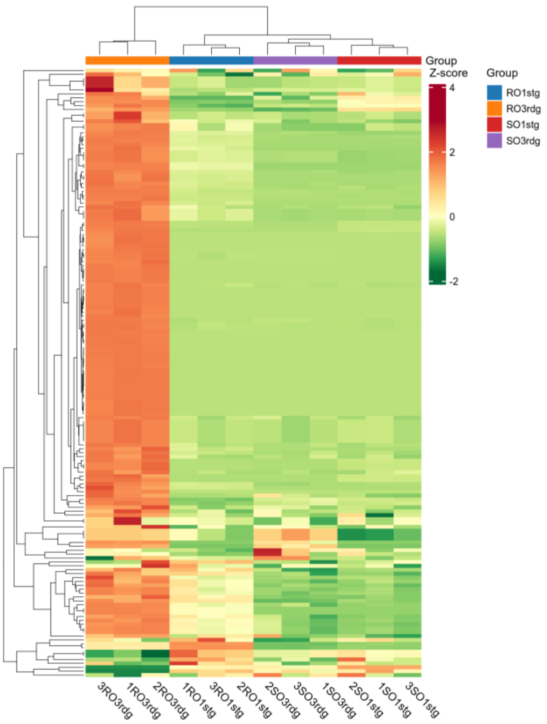

Fig. S1 Heat maps of metabolomics (M), lipidomics (L) and volatileomics (V) illustrating the distribution of metabolites across different treatments in vegetable oil examples. Each sample is represented by a column, while each metabolite is represented by a row. The colors green and red are used to indicate low and high abundance, respectively.
